# Monte Carlo studies on neutron interactions in radiobiological experiments

**DOI:** 10.1371/journal.pone.0181281

**Published:** 2017-07-13

**Authors:** Mehrdad Shahmohammadi Beni, Tak Cheong Hau, D. Krstic, D. Nikezic, K. N. Yu

**Affiliations:** 1 Department of Physics and Materials Science, City University of Hong Kong, Kowloon Tong, Hong Kong, China; 2 Faculty of Science, University of Kragujevac, Kragujevac,Serbia; 3 State Key Laboratory in Marine Pollution, City University of Hong Kong, Kowloon Tong, Hong Kong, China; ENEA Centro Ricerche Casaccia, ITALY

## Abstract

Monte Carlo method was used to study the characteristics of neutron interactions with cells underneath a water medium layer with varying thickness. The following results were obtained. (1) The fractions of neutron interaction with ^1^H, ^12^C, ^14^N and ^16^O nuclei in the cell layer were studied. The fraction with ^1^H increased with increasing medium thickness, while decreased for ^12^C, ^14^N and ^16^O nuclei. The bulges in the interaction fractions with ^12^C, ^14^N and ^16^O nuclei were explained by the resonance spikes in the interaction cross-section data. The interaction fraction decreased in the order: ^1^H > ^16^O > ^12^C > ^14^N. (2) In general, as the medium thickness increased, the number of “interacting neutrons” which exited the medium and then further interacted with the cell layer increased. (3) The area under the angular distributions for “interacting neutrons” decreased with increasing incident neutron energy. Such results would be useful for deciphering the reasons behind discrepancies among existing results in the literature.

## Introduction

The biological effects of neutrons are less well understood compared to other types of ionizing radiations [1]. In particular, there were apparent contradicting results in the literature regarding the neutron-induced bystander effects (NIBEs) and neutron-induced radioadaptive response (RAR). For example, it was noted that neutrons do not produce a bystander effect during the *in vivo* and *in vitro* investigations [2–4]. In contrast, Ng et al. [5] demonstrated the presence of NIBEs in zebrafish embryos when the biological targets (i.e. zebrafish embryos) were irradiated using Neutron exposure Accelerator System for Biological Effect Experiments (NASBEE) facility at the National Institute of Radiological Sciences (NIRS), Chiba, Japan [6]. Furthermore, similar discrepancies were also present in terms of RAR, in which the failure of neutrons to induce RAR in zebrafish embryos and human lymphocytes has been previously reported [7,8] and in contrast, the induction of RAR in Chinese hamster V79 cells was reported in the previous work of Marples and Shov [9].

The experimental conditions (including neutron energies) and the biological targets (including cell lines and organisms, and their states such as the cell cycle phase [10]) employed in the above studies were not identical. In most cases, the thickness of medium layer between the neutron source and the biological target was not controlled, recorded or reported. As such, deciphering the reasons behind these discrepancies would be a challenging task. However, the fundamental parameter remained as the number of biomolecules damaged by the neutrons [11–13] which would be surrogated by the number of neutron interactions (event frequencies) in the targeted cells [14], although these parameters would in turn be determined by the abundance of different atomic nuclei within the targeted cells, as well as the incident neutron energies and the medium thickness etc. [15]. Accordingly, the numbers of neutron interactions or event frequencies in the targeted cells are essential for proper studies and understanding of the biological effects of neutrons, as well as for deciphering the reasons behind discrepancies of reported results. Rossi and Kellerer [10] insightfully remarked that “…*event frequencies in the cell nucleus is crucial to the discussion*, *but has been treated somewhat inadequately and a more rigorous consideration is therefore required*…”. The present work was therefore devoted to a rigorous study on the number of neutron interactions in targeted cells in radiobiological experiments. The main objective was to study four characteristics of neutrons interacting with the cell layer covered with a water medium for different neutron energies. First, the interaction fractions of neutrons with various nuclei (i.e., ratios between number of neutron interactions with specific nuclei and total number of neutron interactions) in the cell layer and the relationships with the medium thickness covering the cell layer were determined. Second, the penetration fractions of neutrons (i.e., ratios between number of neutrons that penetrated through the water-cell system without any interactions and total number of neutrons launched) were assessed. Third, the fractions of neutrons interacting within the medium layer (i.e., ratios between number of neutrons interacting within the medium layer and total number of neutrons that interacted within the water-cell system) were evaluated. Fourth, the angular distributions of neutrons exiting the medium layer and then interacting with cell layer were analyzed.

Three different neutron energies were examined, namely, 100 keV, 2 MeV and 10 MeV. These characteristics would be rigorously revealed through Monte Carlo simulations. A modified version of our previously developed computer program [16] was used to study the neutron transport in the medium layer and the cell layer. The present results and the developed computer program would be useful in determining these neutron characteristics which were inadequately addressed in previous radiobiological experiments. Through such results and tools, it was anticipated that the fundamental parameter, i.e., number of neutron interactions in the targeted cells, for different radiobiological experiments could be realistically assessed, and the reasons behind discrepancies among existing results in the literature could be deciphered.

## Monte Carlo method and NRUneutron code

**Fig 1:**
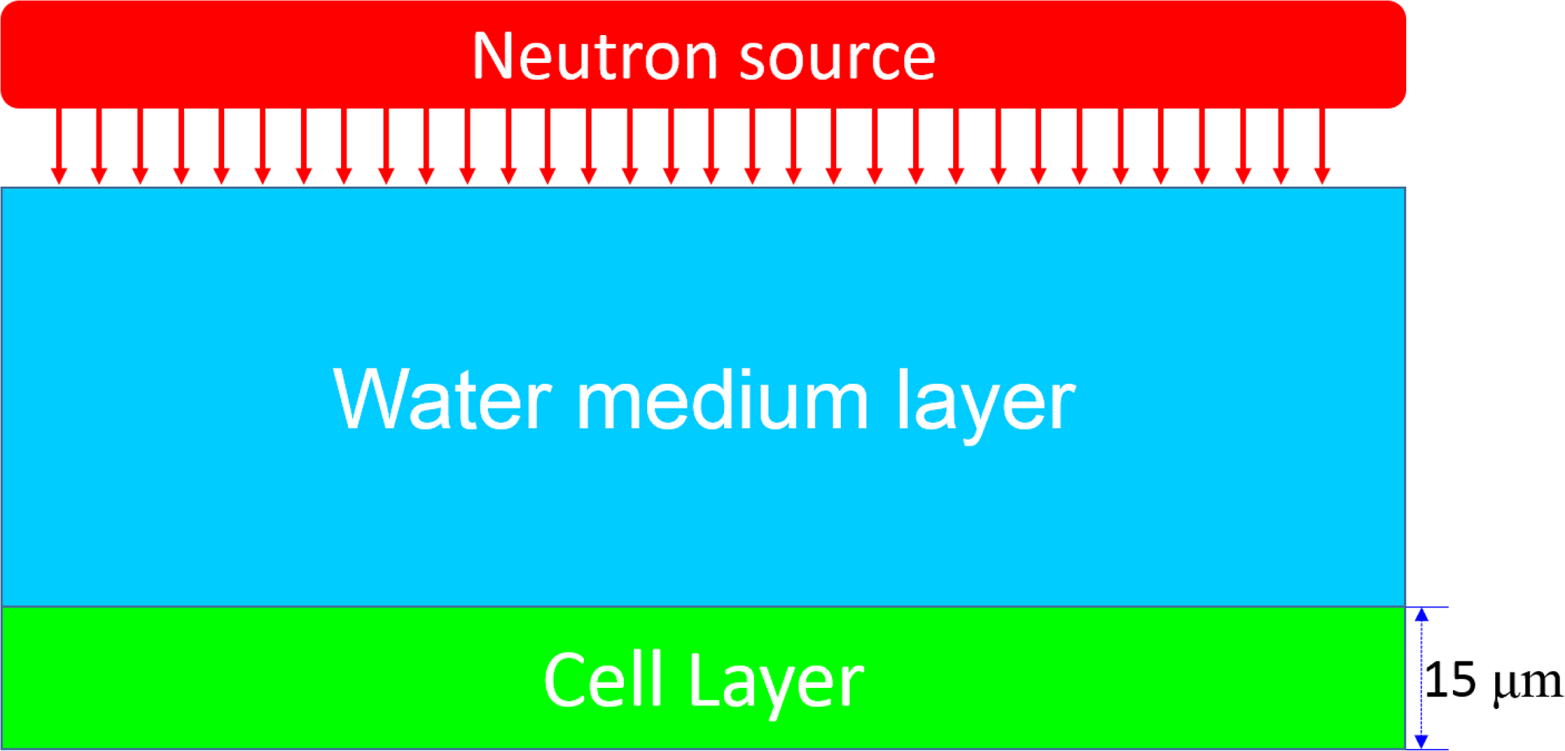
Schematic diagram showing the neutron irradiation setup.

The Monte Carlo method was used to simulate the propagation of neutrons from the water medium layer to the cell layer, and to simulate the neutron interactions with various nuclei in the two layers. The system setup is schematically shown in Fig 1.

**Fig 2:**
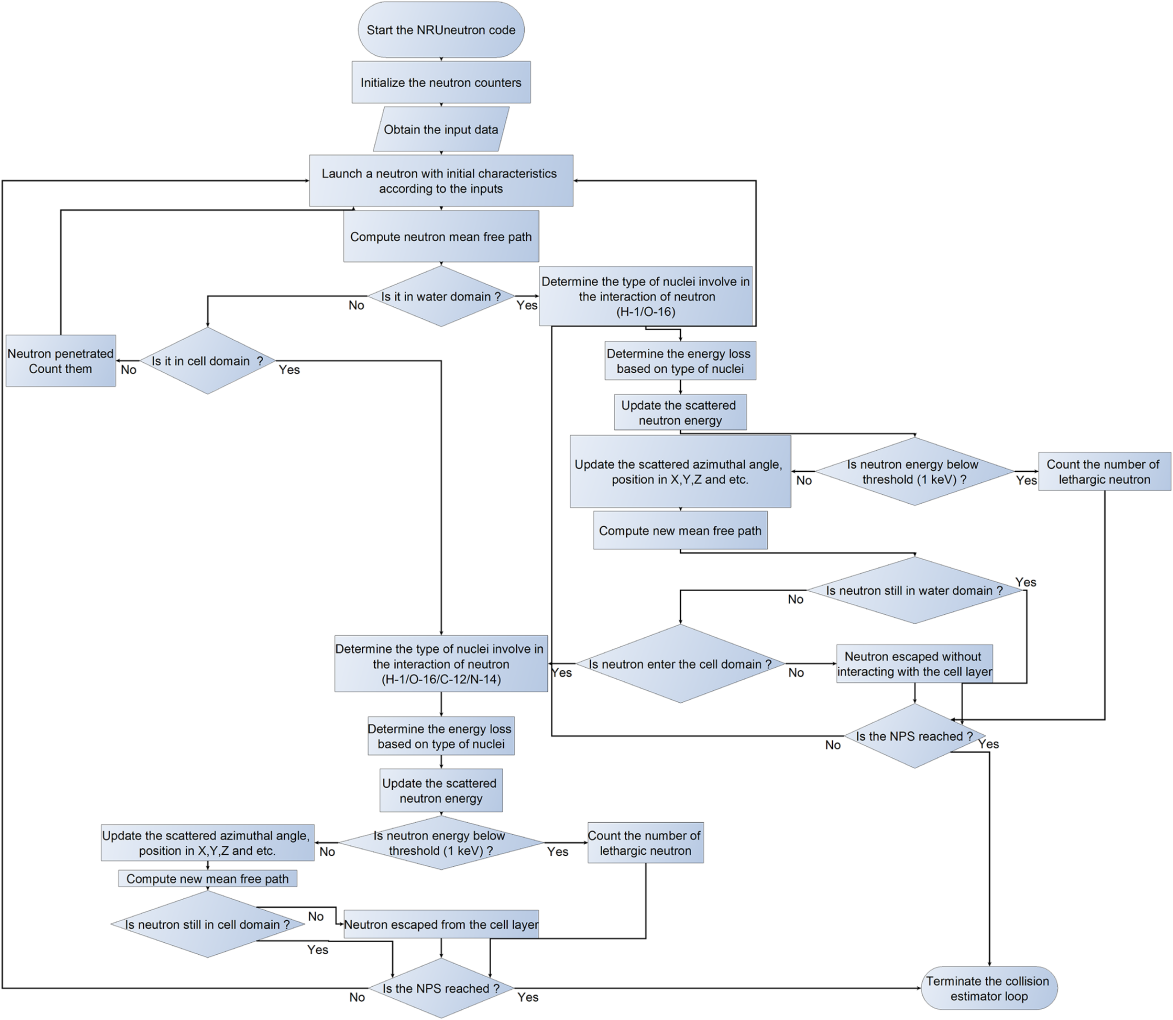
Flowchart for the algorithm in the neutron collision estimator in the NRUneutron code.

The NRUneutron code consisted of two main parts, i.e., (1) the collision estimator and (2) the dose estimator. The collision estimator was built in the main program whereas the dose estimator was a subroutine which was called at the end of the program to read the output energy dissipation of neutrons to compute the doses in the medium and cell layers. A simplified flowchart for the algorithm employed in the collision estimator is shown in Fig 2.

The present computer program was developed using the Fortran90 programming language. Inputs to the present program included (1) neutron energy, (2) total number of neutrons launched (NPS), (3) incident angles and (4) dimensions of the medium and cell-layer domains (*x*, *y*, *z*). The energy dependent neutron cross-section data for different nuclei present in the domains were also required. The water medium domain contained ^1^H and ^16^O while the cell-layer domain was modelled as a tissue equivalent plastic (TEP) which contained ^12^C, ^1^H, ^14^N and ^16^O (density = 1.127 g/cm^3^; mass composition: 11.1% carbon, 10.1% hydrogen, 2.6% nitrogen and 76.2% oxygen). Other appropriate compositions could also be chosen, e.g., from Ref. [17]. It is noted that the culture medium for in vitro experiments in general also contains other constituents (such as proteins etc.) in addition to water. The presence of other constituents in the medium can affect the characteristics of neutrons that interact with the cell layer, which need to be computed for individual scenarios. It would not be feasible to exhaustively summarize here how the obtained results will be changed by the type and amount of constituents in the medium.

## Energy dependent total neutron cross section

**Fig 3:**
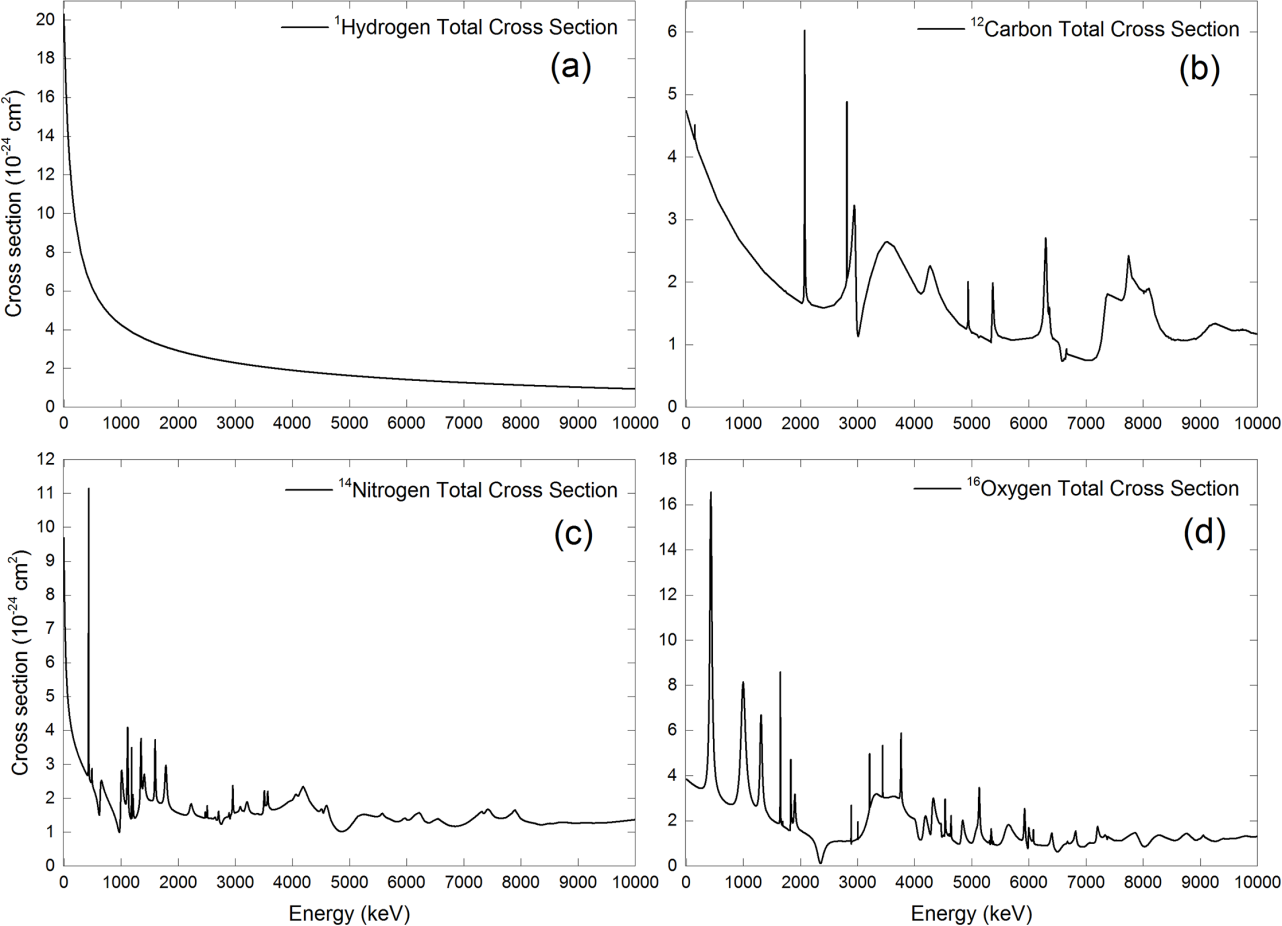
Energy dependent total neutron cross sections for (a) ^1^H, (b) ^12^C, (c) ^14^N and (d) ^16^O nuclei.

The energy dependent neutron cross-section data for different nuclei were adopted from the Evaluated Nuclear Data File) (ENDF) library home page: http://www.nndc.bnl.gov/sigma/tree/index.html. The neutron-energy range from 1 keV to 10 MeV was considered as in our previous work [16]. The cross-section data were interpolated and sorted with an energy increment of 1 keV using the quick 1-D linear interpolation function in MATLAB (ver. R2013a). The total cross-section data used in the present work are shown in Fig 3.

**Fig 4:**
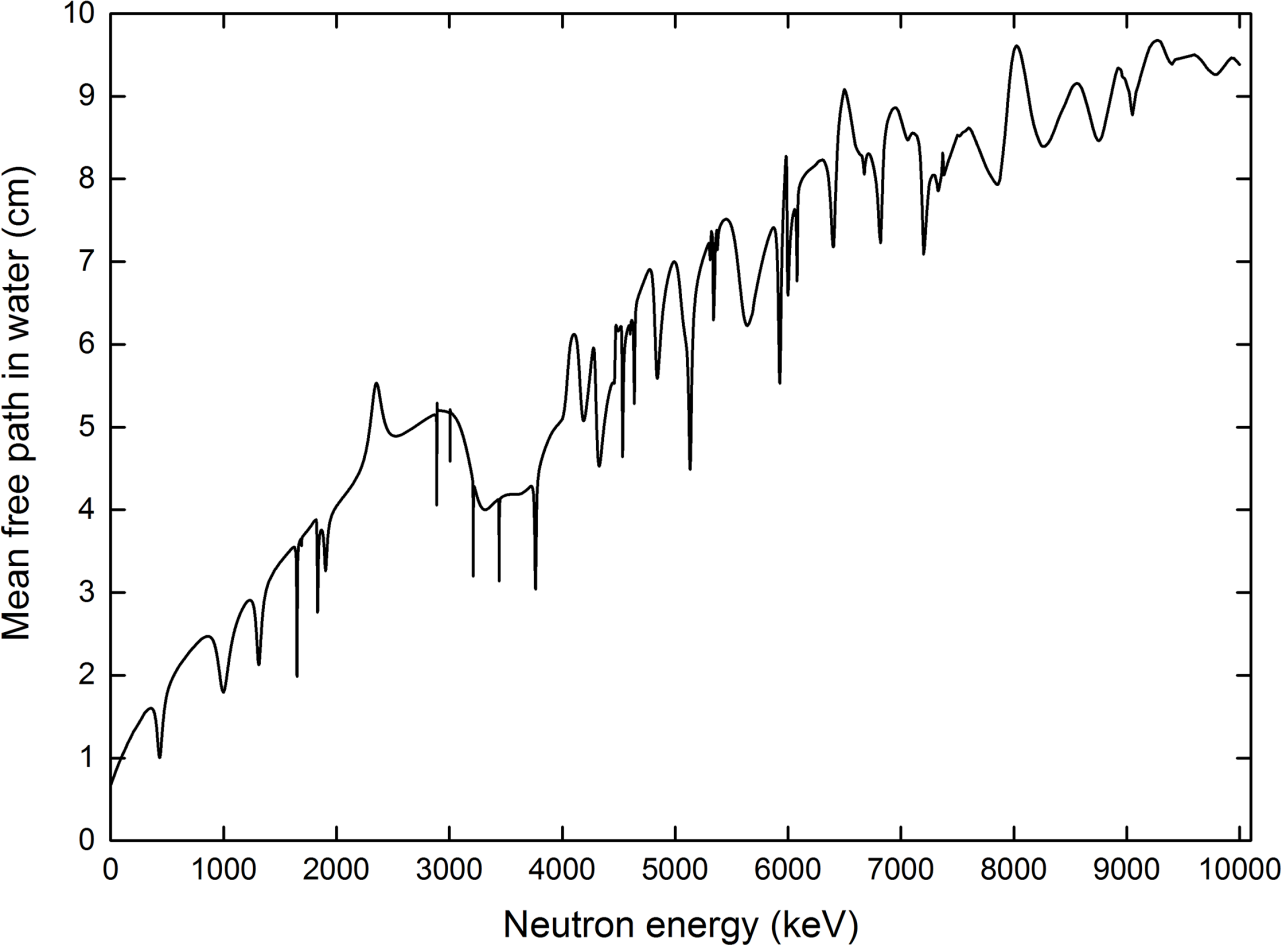
Mean free path for neutrons in the water medium as a function of the neutron energy.

The total macroscopic cross sections (*Ʃ*) for interaction of neutrons with the medium and cell layers are respectively given as:

$$\Sigma_{H_{2}O}=\frac{\rho_{H_{2}O}N_{Av}}{M_{H_{2}O}}(2\sigma_{H,tot}+\sigma_{O,tot})$$ (1)

$$\Sigma_{Cell}=\frac{\rho_{TEP}N_{Av}}{M_{TEP}}(4\sigma_{H,tot}+{4\sigma }_{C,tot}+\sigma_{N,tot}+29\sigma_{O,tot})$$ (2)

where the Avogadro number (*N*_*Av*_) was 6.023×10^23^ mol^-1^, $\rho_{H_{2}O}$ = 1.0 g/cm^3^ and *ρ*_*TEP*_ = 1.127 g/cm^3^, while the molar mass (*M*) for the medium and cell-layer domains were 18 and 530 g/mol, respectively. The coefficient preceding each microscopic cross section (*σ*) was the number of the corresponding nuclei present in a water or TEP molecule. The reciprocal of the macroscopic cross section (*Ʃ*) gave the mean free path, i.e., *λ* = 1/*Ʃ*, which represented the average distance between two successive collisions of a neutron. The computed mean free path of neutrons in the medium domain as a function of neutron energy is shown in Fig 4.

## Geometry and neutron tracking module

**Fig 5:**
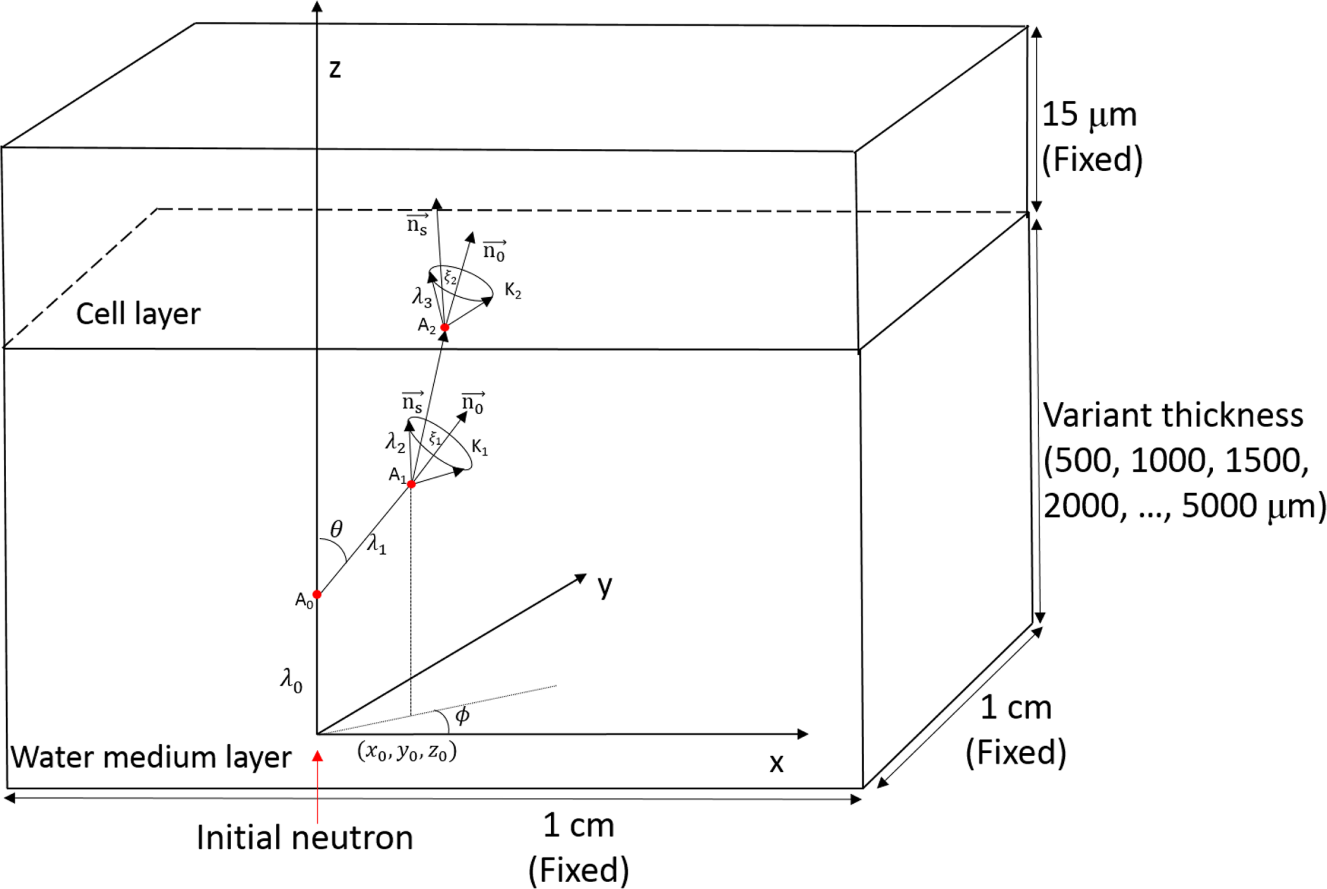
Three-dimensional neutron tracking module and geometry of interaction with water-cell system.

In the present work, the cross-sectional area of the medium and cell layers was chosen to be 1×1 cm^2^ while the thickness for the cell layer was fixed as 15 μm. The monolayer cell thickness is usually around 10–15 μm (see e.g., Ref. [18]). However, variation of cell layer from 10 to 30 μm has negligible effects on the neutron characteristics as shown in our previous study [1] (see Figs 4 and 5 in the Ref. [1]). Ten different medium thicknesses of 500, 1000, 1500, 2000, 2500, 3000, 3500, 4000, 4500 and 5000 μm were studied [1]. We considered that the neutrons initially struck the medium layer perpendicularly at (*x*_0_, *y*_0_, *z*_0_) with an incident energy of *E*_0_. The *z*-axis was defined as the direction of the neutron path from the source towards the medium layer. The neutron tracking module is schematically shown in Fig 5. The first step was to determine the neutron’s initial mean free path (*λ*_0_) through

$$\lambda_{0}=-\frac{1}{\Sigma (E_{0})}\ln\left(\gamma \right)$$ (3)

where *γ* was a uniformly distributed random number in the interval of [0,1].

The domain in which interaction took place was chosen based on the neutron’s mean free path while the nucleus with which the neutron interacted was chosen based on the neutron interaction cross-section data. A neutron with an initial energy of *E*_0_ made its first interaction at the point *A*_0_. Upon interaction with a hydrogen nucleus, the neutron would have its energy reduced to the scattered energy *E*_*sc*_ given by

$$E_{sc}=E_{0}\cdot \gamma$$ (4)

where *γ* was a new random number called through the Fortran90 intrinsic function of CALL RANDOM_NUMBER(GAMMA). After determining *E*_*sc*_, the scattering angle *θ* of the neutron in the laboratory coordinate system was determined as

$$\cos(\theta )=\sqrt{\frac{E_{sc}}{E_{0}}}$$ (5)

The scattering angle *θ* at *A*_0_ defined a cone about the vertical *z*-axis. Accordingly, the angle *ϕ* measured on the *x-y* plane was sampled as *ϕ* = 2*πγ*, where *γ* was another random number. For interactions with other nuclei with atomic mass *A*, *E*_*sc*_ was sampled between *E*_*min*_ and *E*_0_, where *E*_*min*_ = [(*A*–1)/(*A*+1)]^2^*E*_*0*_, whereas the scattering angle *θ* was determined as

$$\cos\theta =\sqrt{\frac{{\left(1+A\right)}^{2}}{4A}\frac{\left(E_{0}-E_{sc}\right)}{E_{0}}}$$ (6)

After interaction at point *A*_0_, the scattered neutron had a new mean free path *λ*_1_ and would interact at point *A*_1_. The program also checked whether the neutron would exit the medium or cell layer (see flowchart in Fig 2). For the interaction at *A*_1_, the scattering angle was *ξ* which defined a cone with the direction of the central axis ($\vec{n_{0}}$) defined by the neutron trajectory before this interaction. The angle *ϕ* was then sampled on a plane containing the circle *K*_1_. These procedures were repeated until the neutron left the layers or completely stopped in the layers.

## Benchmarking of NRUneutron code

**Fig 6:**
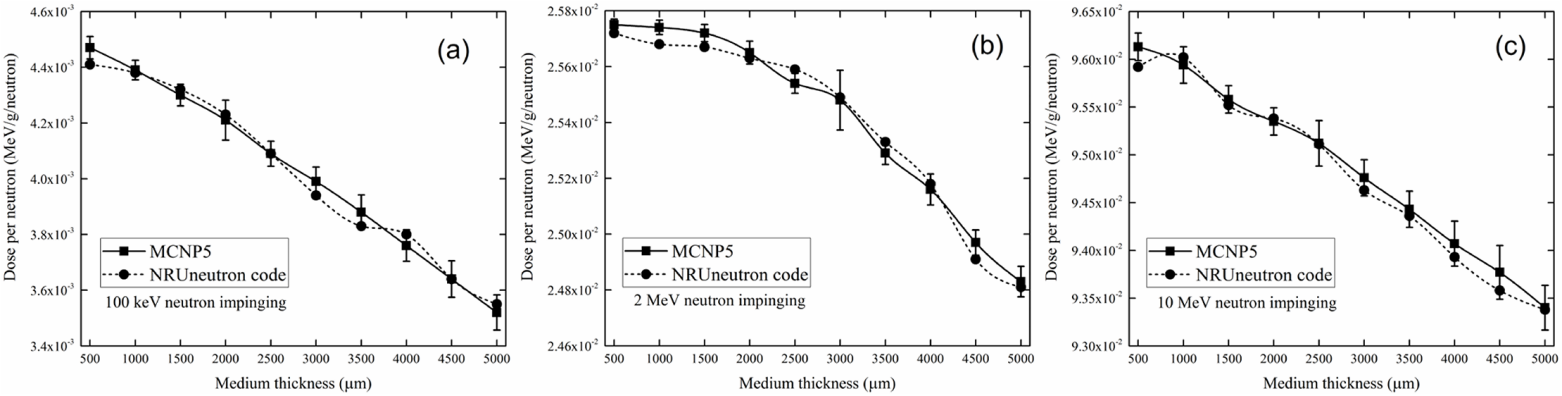
Absorbed neutron doses in the cell layer for different incident neutron energies and medium thicknesses obtained using the MCNP5 and NRUneutron codes. The error bars represent one standard deviations obtained from multiplication of the relative error estimated using the (F6:N) tally by the tally mean value.

The present computer program was benchmarked using the Monte Carlo N-Particle (MCNP) 5 code [19], both of which used pointwise cross-section data. The absorbed neutron dose in the cell layer covered by the water medium was used to compare the reliability and accuracy of the present program. The dose was determined by the interactions along the neutron trajectory within the target volume, so comparisons under different incident neutron energies and medium thicknesses were necessary. The results are shown in Fig 6, which show good agreement. The decrease in the absorbed neutron dose in the cell layer with increasing medium thickness was due to the reduction in neutron energy exiting the thicker medium layer.

## Computation scheme and outputs

The present work focused on the effects of the incident neutron energy (namely, 100 keV, 2 and 10 MeV) and water medium thickness on the absorbed neutron dose, so we kept the cell-layer thickness constant at 15 μm. The output results were:

1. Interaction fraction of neutrons with ^1^H, ^12^C, ^14^N and ^16^O nuclei within cell layer;
2. Penetration fraction of neutrons;
3. Fraction of neutrons interacting within medium layer; and
4. Angular distribution of neutrons exiting medium layer and then interacting with cell layer.

## Interaction fraction of neutrons with various nuclei within cell layer

**Fig 7:**
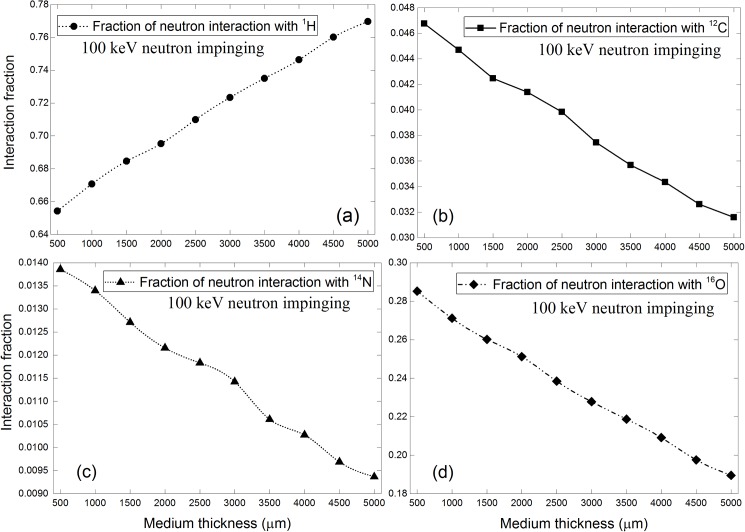
Interaction fractions of neutrons with (a) ^1^H, (b) ^12^C, (c) ^14^N and (d) ^16^O within cell layer for 100 keV incident neutrons.

The results for 100 keV incident neutrons are shown in Fig 7. The interaction fractions decreased with increasing medium layer thickness for ^12^C, ^14^N and ^16^O nuclei, but increased with increasing medium thickness for ^1^H nuclei. As shown in Fig 3, the interaction cross sections with ^1^H nuclei were much larger compared to other nuclei, so the neutron interactions would be dominated by those with ^1^H nuclei. A thicker medium layer absorbed more energy from a neutron and thus significantly increased its interaction probability with ^1^H nuclei, so the interaction fraction with ^1^H nuclei within the cell layer increased with increasing medium thickness. Despite that in our computations only 10.1% of the cell content (approximated using TEP material) was made up of hydrogen, interaction fractions with ^1^H nuclei were the largest due to its larger interaction cross sections. In relation, the increase in the interaction fractions with ^1^H nuclei reduced the chance of neutron interactions with ^12^C, ^14^N and ^16^O nuclei so the interaction fractions with these nuclei decreased with increasing medium thickness. The decrease in the interaction fractions in the order ^16^O > ^12^C > ^14^N nuclei was mainly due to the relative abundance of these nuclei in the cell layer, namely, ^16^O (76.2%), ^12^C (11.1%) and ^14^N (2.6%).

**Fig 8:**
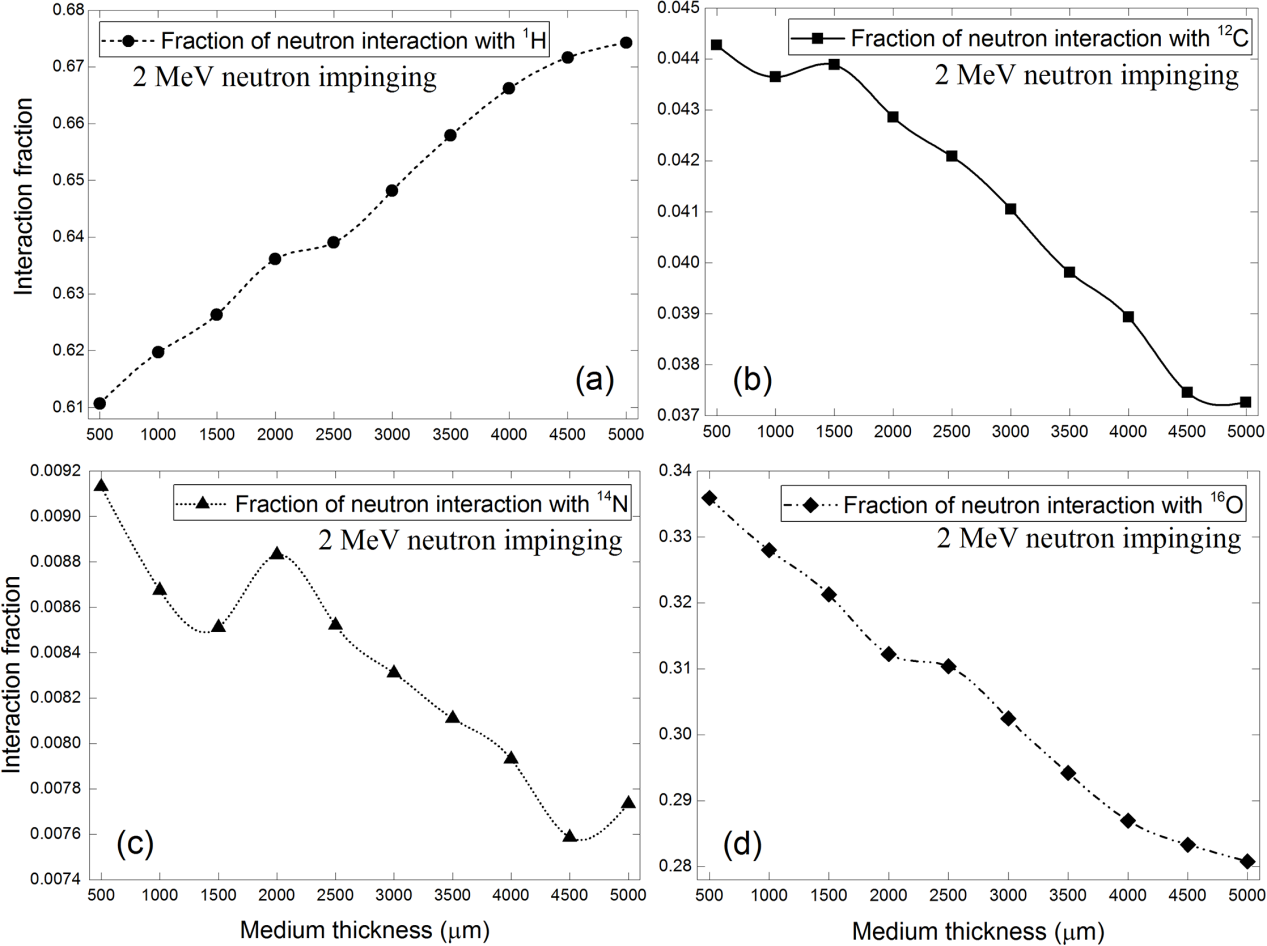
Interaction fractions of neutrons with (a) ^1^H, (b) ^12^C, (c) ^14^N and (d) ^16^O within cell layer for 2 MeV incident neutrons.

The corresponding results for 2 MeV incident neutrons are shown in Fig 8, and the trends were similar to those for 100 keV incident neutrons. The decrease in the interaction fraction in the order: ^1^H > ^16^O > ^12^C > ^14^N remained the same. The magnitudes of interaction fractions were different between 100 keV and 2 MeV incident neutrons due to the different interaction cross sections. In particular, the interaction fractions with ^1^H nuclei were reduced when compared to 100 keV incident neutrons due to reduced interaction cross sections for 2 MeV neutrons (see Fig 3). The main new features here were the conspicuous bulges in the interaction fractions with ^12^C, ^14^N and ^16^O nuclei, which were due to the resonances in the interaction cross sections for ^12^C, ^14^N and ^16^O nuclei for neutron energies larger than 100 keV as shown in Fig 3. As a result of energy reduction of neutrons (initially launched at 2 MeV) while traversing the medium layer, their interaction cross sections would fall into resonance regions which significantly enhanced their interaction probabilities.

**Fig 9:**
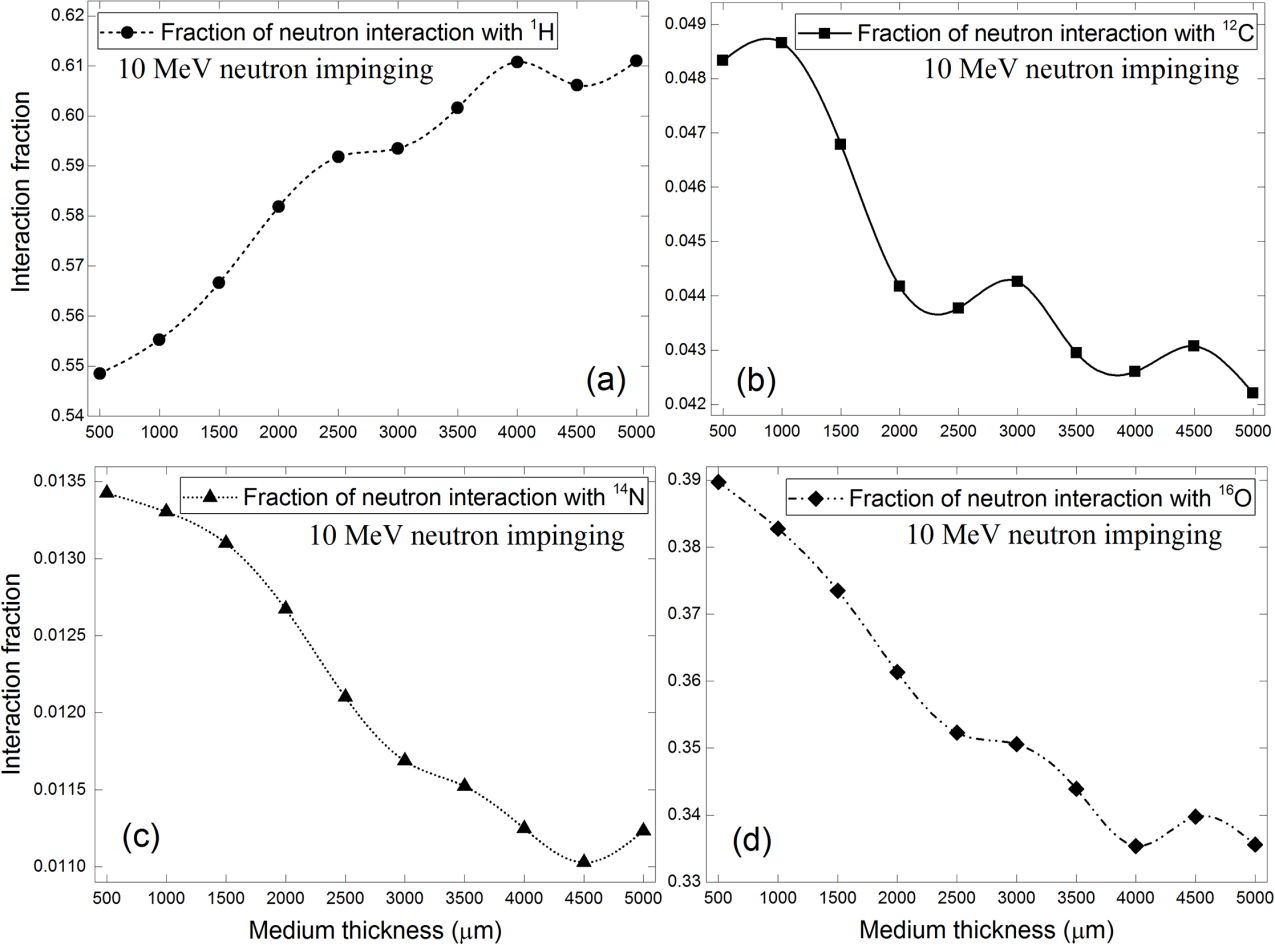
Interaction fractions of neutrons with (a) ^1^H, (b) ^12^C, (c) ^14^N and (d) ^16^O within cell layer for 10 MeV incident neutrons.

The corresponding results for 10 MeV incident neutrons are shown in Fig 9, and the general trends were similar to those for 100 keV and 2 MeV incident neutrons. The decrease in the interaction fraction in the order: ^1^H > ^16^O > ^12^C > ^14^N remained the same. The magnitudes of interaction fractions were changed as a result of different interaction cross sections. The interaction fractions with ^1^H nuclei were further reduced when compared to 2 MeV incident neutrons due to reduced interaction cross sections for 10 MeV neutrons (see Fig 3). The interaction fractions for 10 MeV neutrons with ^12^C nuclei displayed more conspicuous bulges compared to other nuclei, which was explained by the resonance spikes in its interaction cross sections for energies below 10 MeV.

## Penetration fraction of neutrons

**Fig 10:**
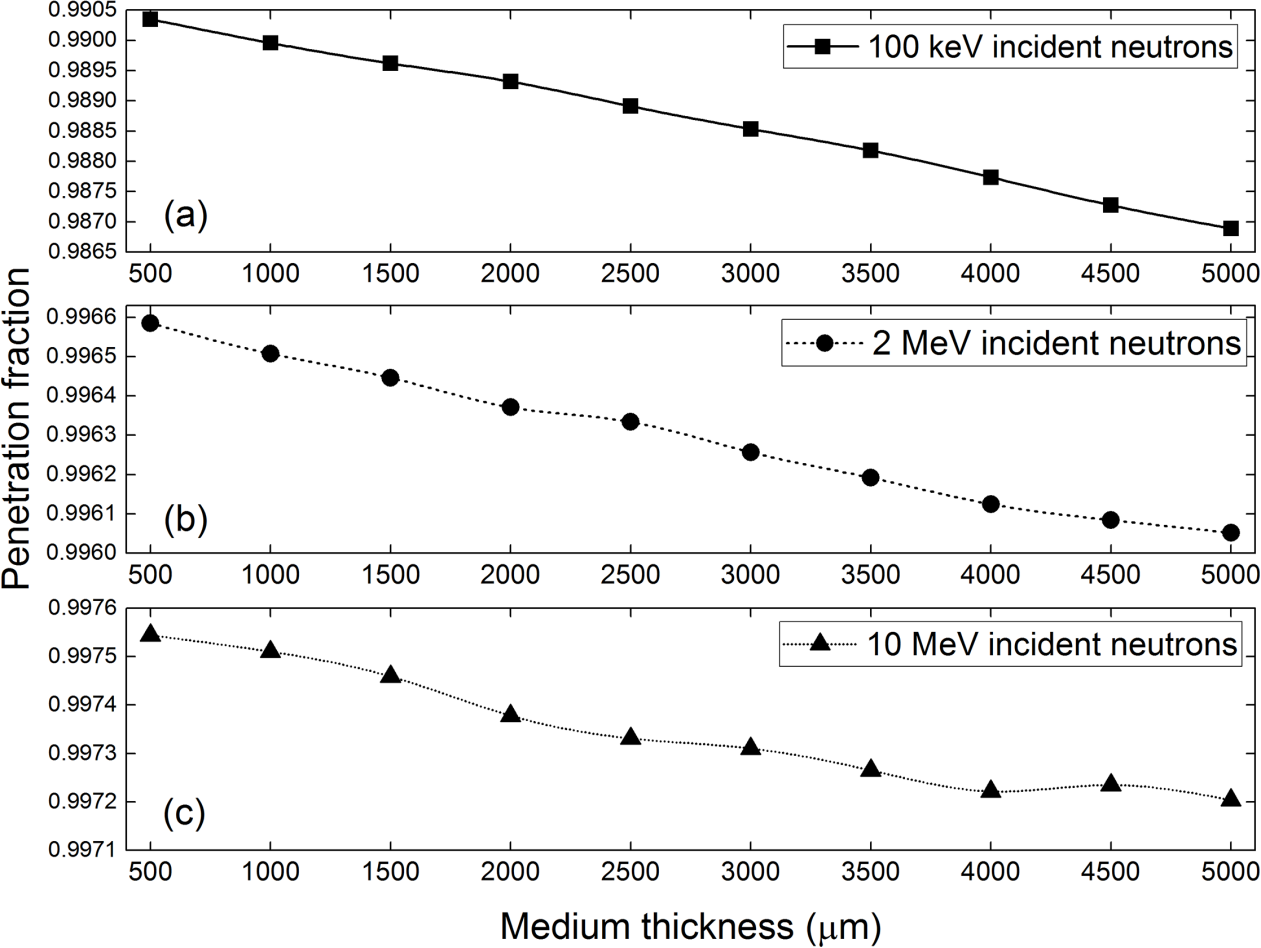
Penetration fractions of neutrons from the cell-medium system for neutrons with incident energies of (a) 100 keV, (b) 2 MeV and (c) 10 MeV.

The penetration fractions of neutrons with incident energies of 100 keV, 2 and 10 MeV are shown in Fig 10. As expected, the penetration fractions decreased with increasing medium thickness, since a thicker medium contained a larger number of atoms so the probability of having a neutron interaction would be higher. In our previous work, a similar trend was obtained for the penetration fraction of neutrons through a polyethylene layer [16]. On the other hand, the penetration fraction increased with the incident neutron energy, which was explained by the corresponding reduction in the interaction cross sections. For higher incident neutron energies, the effect of medium thickness on the penetration fraction was diminished.

## Fraction of neutrons interacting within medium layer

**Fig 11:**
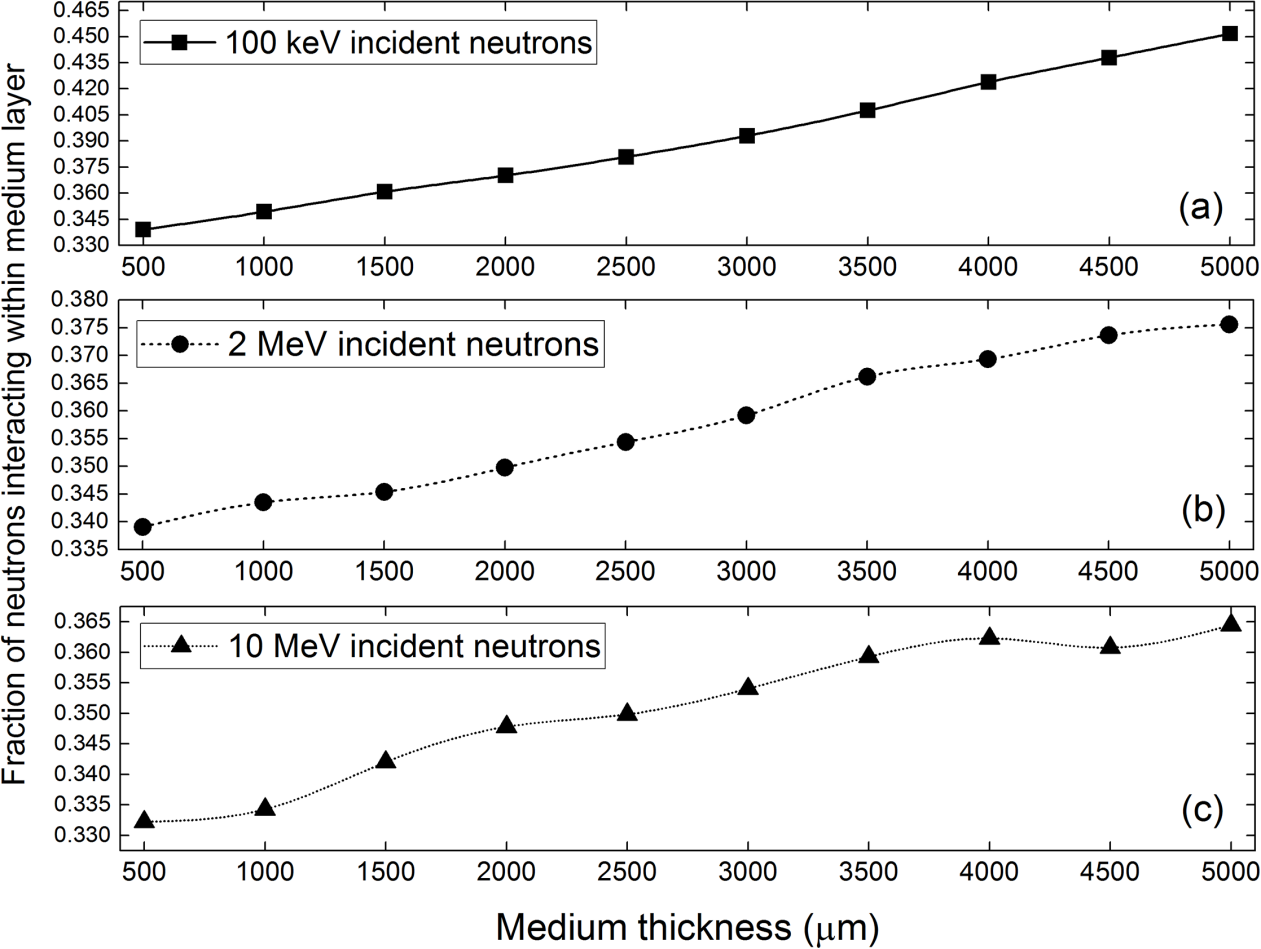
Fractions of neutrons interacting within medium layer at (a) 100 keV, (b) 2 MeV and (c) 10 MeV incident neutron energy.

The fractions of neutrons interacting within the medium layer are shown in Fig 11 to increase with the medium thickness, which is expected since thicker media contained more nuclei to enable more effective neutron interactions. Fig 11 also shows that these fractions decrease with increasing incident neutron energy. An interesting observation was the absence of conspicuous bulges in the trend for 2 MeV neutrons, despite the large and dense resonance spikes in the interaction cross sections of ^16^O in the 2-MeV region, which was explained by the insufficient shifting of the neutron energy within the medium layer. To achieve significant effects from the resonance spikes in the interaction cross sections, the neutron energy should have been adequately shifted to reach the resonance spikes in the interaction cross sections, which meant that the neutrons should have traversed the entire medium layer. Therefore, when the medium layers were studied alone, the effect of resonances was negligible. Furthermore, the minimum and maximum fractions shown in Fig 11(A) to 11(C) were ~33% and 46%, respectively. Accordingly, the fractions of neutrons interacting within the cell layer would be larger (minimum of ~54% and maximum of ~67%; not counting those neutrons penetrating through the cell-medium system without interactions here), which was explained by the reduction of neutron energy through the medium layer and the corresponding increase in the probability of neutron interaction in the cell layer.

## Angular distributions of neutrons exiting medium layer and then interacting with cell layer

**Fig 12:**
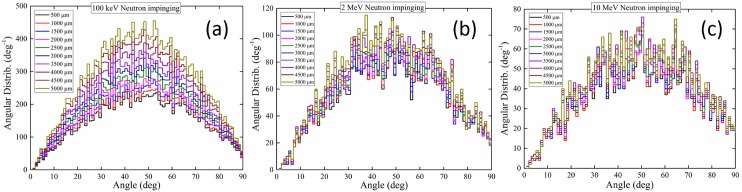
Angular distributions of neutrons exiting the medium layer that interact in the cell layer for incident neutron energies of (a) 100 keV, (b) 2 MeV and (c) 10 MeV.

The angular distributions (*dn*/*dθ*) of neutrons exiting medium layer and then interacting with cell layer (hereafter referred to as the angular distributions) are shown in Fig 12. It is remarked that those neutrons which have penetrated through the cell layer without interactions after exiting the medium layer are not included in these distributions. The areas under the angular distributions shown in Fig 12, i.e., ($\int_{\theta_{min}}^{\theta_{max}}\frac{dn}{d\theta }d\theta$), represent the total numbers of neutrons (“interacting neutrons”) which have exited from the medium layer and then have undergone interactions in the underlying cell layer. For neutrons perpendicularly impinging the medium layer, a peak in the angular distributions was generally observed from 50° to 55°. As the neutron energy increased, this peak shifted toward angles larger than ~45° mainly due to scattering of neutrons with ^1^H nuclei, which is dominating the neutron interaction. Incidentally, an associated scattering phenomenon was noted in our previous work [16] where the peak in the angular distributions of ejected protons would be smaller than ~45° when neutrons perpendicularly impinged the target. The sum of scattering angles of the neutron and ^1^H nucleus was 90° in the laboratory frame. In Eq (5), the term *E*_*sc*_*/E*_*0*_ was always smaller than unity. As the neutron lost more energy during its propagation in the medium layer, its scattering angle would be larger than ~45°.

**Fig 13:**
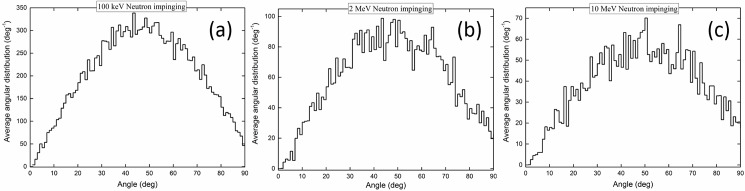
Average angular distributions of neutrons exiting the medium layer that interact in the cell layer for incident neutron energies of (a) 100 keV, (b) 2 MeV and (c) 10 MeV.

For the incident neutron energy of 100 keV, the number of interacting neutrons increased with the medium thickness. The same trend was also observed for higher incident neutron energies of 2 and 10 MeV, but with weaker dependence on the medium thickness. The phenomenon was expected as an increase in the medium thickness would reduce the neutron energy and the exited neutron could more likely undergo interactions within the underlying cell layer. It was also noted that the total number of “interacting neutrons” in the cell layer (area under the angular distributions) decreased with increasing incident neutron energy. In order to have a more informative comparison, the average angular distributions (averaged for different medium thicknesses) for specific incident neutron energies were plotted, which are shown in Fig 13. For an equal number of neutrons entering the medium layer, the number of neutrons exiting from the medium layer and then underwent some interactions in the underlying cell layer for incident neutron energy of 100 keV was ~3.4 times higher than the corresponding number for incident neutron energy of 2 MeV, which was in turn ~1.5 times higher than the corresponding number for incident neutron energy of 10 MeV.

## Conclusions and discussion

The characteristics and the underlying mechanism of neutron interactions during radiobiological experiments were comprehensively investigated using the self-written NRUneutron computer code. The reliability of the obtained results was assessed by benchmarking the absorbed neutron dose in the cells underneath the medium layer with varying thicknesses. A number of important observations were made:

1. The interaction fraction of neutrons with ^1^H nuclei within the cell layer increased with the medium thickness, whereas the interaction fractions with ^12^C, ^14^N and ^16^O nuclei decreased with increasing medium thickness. The conspicuous bulges in the interaction fractions with ^12^C, ^14^N and ^16^O nuclei were explained by the resonance spikes in the interaction cross-section data, mainly for neutron energies larger than 100 keV. The interaction fraction of neutrons decreased in the order: ^1^H > ^16^O > ^12^C > ^14^N.
2. In general, as the medium thickness increased, the number of neutrons which exited the medium and then further interacted with the cell layer increased.
3. The area under the angular distributions for “interacting neutrons” decreased with increasing incident neutron energy. For an equal number of neutrons entering the medium layer, the number of interacting neutrons for incident neutron energy of 100 keV was ~3.4 times higher than the corresponding number for incident neutron energy of 2 MeV, which was in turn ~1.5 times higher than the corresponding number for incident neutron energy of 10 MeV.

These results highlighted the critical dependence of the number of neutron interactions in the targeted cells, which was a fundamental parameter controlling the radiobiological effects of neutron irradiation, on the experimental conditions (neutron energies and medium thickness) and the biological targets themselves (abundance of different atomic nuclei within the targeted cells). As such, special attention needs to be paid to comparisons among different experimental results obtained using different neutron energies.

The developed computer program would be useful in determining these neutron characteristics and would be made public. It is hoped that in all future studies on radiobiological effects of neutrons, these characteristics of neutron interactions could be obtained using the program and reported to enable more meaningful comparisons. It is also hoped that such comparisons will avoid further discrepancies in the results. Notwithstanding, it is also noted that such discrepancies might also be due to biological reasons, e.g., different cells might respond to radiation in different ways.
